# Pectolinarigenin from *Tiliacora triandra* Exhibits Potent Anticancer Activity in Triple-Negative Breast Cancer Cells Through Cell Cycle Arrest, Apoptosis, and MAPK Signaling Inhibition

**DOI:** 10.3390/ph19030384

**Published:** 2026-02-27

**Authors:** Punnida Arjsri, Warathit Semmarath, Kamonwan Srisawad, Intranee Intanil, Pilaiporn Thippraphan, Pornngarm Dejkriengkraikul

**Affiliations:** 1 Department of Biochemistry, Faculty of Medicine, Chiang Mai University, Chiang Mai 50200, Thailand; punnida.dream@gmail.com (P.A.); k.srisawad@gmail.com (K.S.); intranee.in@cmu.ac.th (I.I.); tipprapant@gmail.com (P.T.); 2 Akkhraratchakumari Veterinary College, Walailak University, Nakhon Si Thammarat 80160, Thailand; 3 One Health Research Center, Walailak University, Nakhon Si Thammarat 80160, Thailand; 4 Anticarcinogenesis and Apoptosis Research Cluster, Faculty of Medicine, Chiang Mai University, Chiang Mai 50200, Thailand; 5 Center for Research and Development of Natural Products for Health, Chiang Mai University, Chiang Mai 50200, Thailand

**Keywords:** *Tiliacora triandra*, pectolinarigenin, triple-negative breast cancer, natural products, cell cycle arrest, apoptosis, MAPK signaling

## Abstract

**Background/Objectives:** Breast cancer is the most commonly diagnosed cancer among women worldwide, with triple-negative breast cancer (TNBC) being a highly aggressive subtype characterized by early recurrence, limited targeted therapies, and poor clinical outcomes. Despite advances in chemotherapy, therapeutic resistance remains a major challenge, underscoring the need for alternative therapeutic approaches. Natural products continue to serve as important sources of bioactive compounds for cancer drug discovery. *Tiliacora triandra*, a Thai medicinal plant traditionally used to manage inflammatory and metabolic disorders, has not been extensively investigated for its potential against TNBC. In this study, we evaluated the anti-cancer effects of *T. triandra* extracts and its major flavonoid constituent, pectolinarigenin, in triple-negative breast cancer, MDA-MB-231 cells. **Methods:** An 80% ethanolic root extract was sequentially partitioned into hexane, dichloromethane, and ethyl acetate fractions. High-performance liquid chromatography identified pectolinarigenin as a predominant component of the dichloromethane fraction (TT-DCM), with a quantified content of 14.24 ± 2.32 mg/g extract. The anti-cancer effect of TT-DCM and pectolinarigenin on MDA-MB-231 cells were investigated using colony formation, cell cycle analysis, PI/Annexin V staining, and Western blot analysis. **Results:** Both TT-DCM and pectolinarigenin significantly reduced MDA-MB-231 cell viability and clonogenic growth. Treatment resulted in G0/G1 phase accumulation, accompanied by decreased expression of cyclin D1, CDK2, and CDK4. Apoptotic induction was observed, as evidenced by lower expression levels of Bcl-xL, Bcl-2, and surviving proteins, together with increased caspase-9 and caspase-3 activities. Additionally, TT-DCM and pectolinarigenin were associated with reduced phosphorylation of ERK1/2, JNK1/2, and p38 MAPKs. **Conclusions:** Collectively, these findings demonstrate that pectolinarigenin derived from *T. triandra* exerts potent anti-cancer activity in MDA-MB-231 TNBC cells through coordinated modulation of cell cycle progression, apoptotic signaling, and MAPK pathway activity. Further studies are warranted to validate these effects in additional TNBC models.

## 1. Introduction

Breast cancer remains the most frequently diagnosed malignancy among women worldwide, with approximately 2.3 million new cases reported in 2022. It is also a leading cause of cancer-related mortality, accounting for nearly 670,000 deaths per year [1]. In Thailand, breast cancer constitutes the most prevalent female malignancy, with an incident of 37.8 per 100,000 population and more than 22,000 newly diagnosed cases reported in 2020. Mortality rates remain substantial, underscoring the ongoing clinical burden of this disease [2]. Among breast cancer subtypes, Triple-negative breast cancer (TNBC) accounts for approximately 15–20% of breast cancer cases and is characterized by the absence of estrogen receptor (ER), progesterone receptor (PR), and human epidermal growth factor receptor 2 (HER2) expression. This molecular profile limits the effectiveness of targeted therapies and contributes to the aggressive clinical behavior of TNBC, characterized by early recurrence, high metastatic potential, and poor prognosis despite intensive treatment [3,4,5].

Systemic management of TNBC continues to rely primarily on cytotoxic chemotherapy. While initial tumor regression is often observed, treatment outcomes are frequently compromised by dose-limiting toxicity and the rapid emergence of drug resistance [5,6]. Consequently, durable therapeutic responses remain difficult to achieve, highlighting the need for novel agents capable of targeting TNBC through alternative molecular mechanisms with improved safety profiles. Natural products have historically provided a foundation for anti-cancer drug development, exemplified by clinically approved agents such as paclitaxel, vinca alkaloids, and camptothecin derivatives [7,8,9]. Increasing attention has been directed toward plant-derived compounds that exhibit multi-targeted biological activities, including regulation of cell proliferation, apoptosis, and oncogenic signaling networks. Such phytochemicals offer attractive opportunities for the discovery of new anticancer therapeutics, particularly for malignancies with limited treatment options, including TNBC [10,11].

*Tiliacora triandra* (Colebr.) Diels is a medicinal plant frequently used in Southeast Asia for controlling of fever, gastrointestinal disorders, metabolic conditions, and inflammatory diseases [12,13]. Phytochemical studies have identified diverse bioactive constituents within *T. triandra*, including flavonoids and alkaloids such as pectolinarigenin [14] and tiliacorinine [15], which have demonstrated antioxidant and anti-inflammatory activities. Pectolinarigenin, a naturally occurring flavonoid, has attracted growing interest due to its reported biological effects across multiple disease models. Previous studies have shown that pectolinarigenin suppresses proliferation in bladder carcinoma cells through modulation of DNA-damage and autophagy pathways, attenuates inflammatory responses via NF-kB/Nrf2 signaling, alleviates oxidative stress in renal injury models through regulation of hypoxia-inducible factor-1α [16], and inhibits osteosarcoma cell growth by affecting JAK/STAT signaling [17]. Despite these promising findings, the therapeutic potential of *T. triandra* extracts and pectolinarigenin in TNBC has not yet been systematically investigated.

Aberrant activation of intracellular signaling pathways plays a pivotal role in TNBC progression, with the mitogen-activated protein kinase (MAPK) network emerging as a core regulator of tumor cell proliferation, survival, and therapeutic resistance [18,19]. The MAPK cascade comprises three principal kinase modules: extracellular-signal-regulated kinases (ERK1/2), c-Jun N-terminal kinases (JNK1/2), and p38 MAPKs, which integrate extracellular stimuli such as growth factors, inflammatory mediators, and cellular stress signals to control transcriptional programs governing cell cycle dynamics and apoptotic responses. Dysregulated MAPK signaling has been frequently observed in TNBC and is increasingly recognized as a viable pharmacological target [20,21]. Compounds capable of modulating this pathway may therefore provide meaningful therapeutic benefits.

In this study, we investigated the anti-cancer potential of the dichloromethane fraction of *Tiliacora triandra* (TT-DCM) and its bioactive constituent, pectolinarigenin, in MDA-MB-231 triple-negative breast cancer cells. We assessed their effects on cell viability, clonogenic growth, cell cycle progression, and apoptotic signaling, with particular em-phasis on MAPK-associated molecular pathways. Collectively, our results demonstrate that TT-DCM and pectolinarigenin exert significant anti-proliferative and pro-apoptotic effects in this model, highlighting their potential as promising candidates for the development of alternative or adjunctive therapeutic strategies.

## 2. Results

### 2.1. Phytochemical Characterization of T. triandra Extracts

Total phenolic and total flavonoid contents were quantified in the *T. triandra* preparations (TT-EtOH, TT-DCM, TT-EA, and TT-H_2_O). Among these fractions, TT-EA exhibited the highest TPC and TFC (231.40 ± 28.86 mg GAE/g extract and 110.95 ± 5.34 mg CE/g extract, respectively; Table 1). TT-DCM demonstrated the second-highest TFC (101.14 ± 5.91 mg CE/g extract).

**Figure 1:**
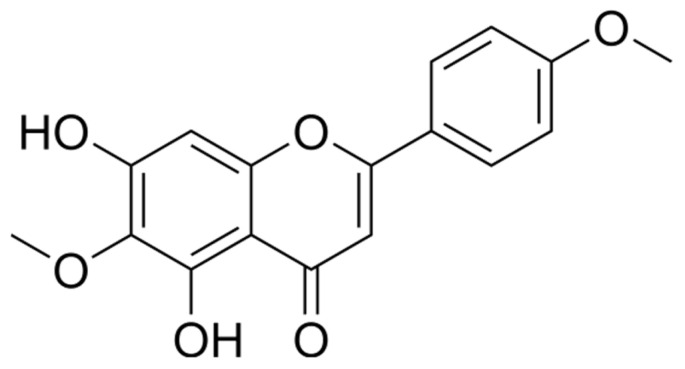
Chemical structure of pectolinarigenin.

High-performance liquid chromatography (HPLC) analysis was performed to quantify pectolinarigenin (chemical structure shown in Figure 1). Compound identification was established by (i) matching retention time with an authentic pectolinarigenin standard analyzed under identical chromatographic conditions, (ii) co-injection of TT-DCM with the reference standard, which produced a single enhanced peak without evidence of peak splitting, and (iii) consistent UV absorption characteristics monitored at 331 nm. Representative chromatograms of the standard, TT-DCM sample, and co-injection experiment are provided in Supplementary Figure S1.

Quantitative analysis revealed that TT-DCM contained the highest level of pectolinarigenin (14.24 ± 2.32 mg/g extract), whereas TT-EA and TT-H_2_O showed no detectable pectolinarigenin under the same analytical conditions (Table 1). The relatively high flavonoid content observed in TT-DCM is consistent with the enrichment of this flavonoid marker in the dichloromethane fraction. Collectively, these findings identify TT-DCM as the fraction most enriched in pectolinarigenin and support its selection for subsequent biological investigations alongside the purified compound.

### 2.2. Effects of T. triandra Extracts and Pectolinarigenin on MDA-MB-231 Cell Viability

**Figure 2:**
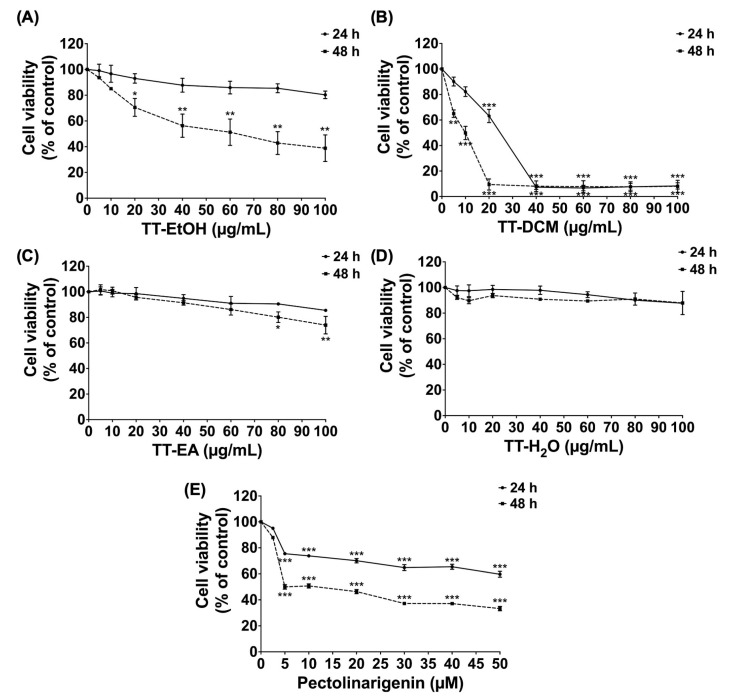
Effects of *T. triandra* extracts and pectolinarigenin on the viability of MDA-MB-231 cells using SRB assay. The cells were pre-treated with TT-EtOH (**A**), TT-DCM (**B**), TT-EA (**C**), TT-H_2_O (**D**) at concentrations of 0–100 µg/mL, or to pectolinarigenin (**E**) at 0–50 µM for 24 and 48 h. Results are shown as mean ± S.D. from three independent experiments. Statistical significance is indicted as * *p* < 0.05, ** *p* < 0.01, and *** *p* < 0.001.

The cytotoxicity of *T. triandra* extracts/fractions and pectolinarigenin was examined in MDA-MB-231 cells using the SRB assay at 24 and 48 h (Figure 2). Among the crude extract and fractions, TT-DCM showed the highest potency, with an IC_50_ of 10.73 ± 2.84 μg/mL at 48 h. TT-EtOH exhibited lower cytotoxicity (IC_50_ = 62.25 ± 5.68 μg/mL at 48 h). In contrast, TT-EA and TT-H_2_O did not reduce cell viability within the tested concentration range (0–100 μg/mL) at 48 h.

Purified pectolinarigenin also decreased MDA-MB-231 cell viability, with an IC_50_ of 5.36 ± 1.02 μM. Based on (i) the higher cytotoxicity potency of TT-DCM and (ii) its elevated pectolinarigenin content, TT-DCM was selected for downstream mechanistic experiments in parallel with pectolinarigenin.

### 2.3. TT-DCM and Pectolinarigenin Reduce Clonogenic Growth of MDA-MB-231 Cells

**Figure 3:**
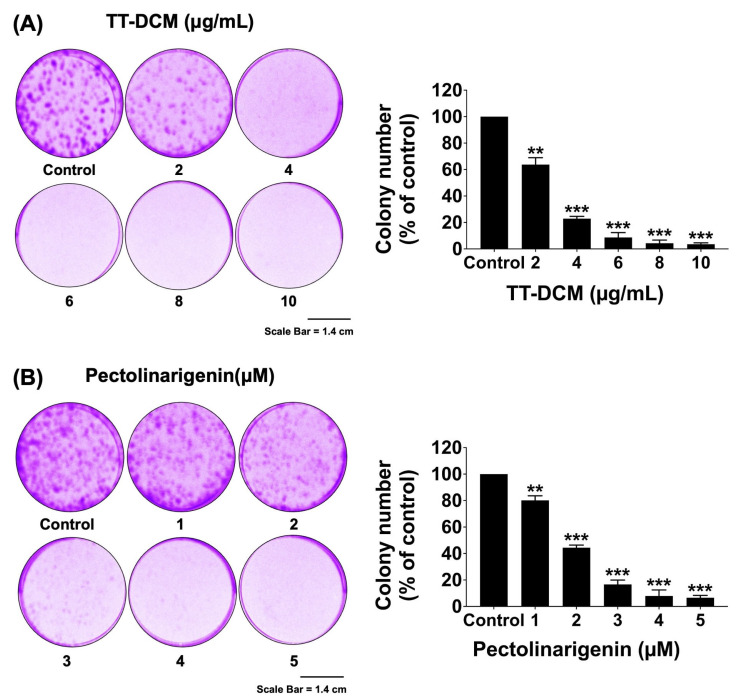
Effect of TT-DCM and pectolinarigenin on clonogenic growth of MDA-MB-231 cells. MDA-MB-231 cells were incubated with TT-DCM (0–10 µg/mL; (**A**)) or pectolinarigenin (0–5 µM; (**B**)) for 7 days, followed by assessment of colony formation. Colonies were visualized by phase-contrast microscopy and calculated using IMAGE J software v.1.140. Results are shown as mean ± S.D. from three independent experiments. Statistical significance vs. untreated controls is indicated as ** *p* < 0.01, and *** *p* < 0.001.

To assess long-term proliferative capacity, clonogenic assays were performed following continuous exposure to TT-DCM (0–10 µg/mL) or pectolinarigenin (0–5 µM) for 7 days. TT-DCM significantly decreased colony formation in a concentration-dependent manner (*p* < 0.001; Figure 3A). A comparable reduction in clonogenic survival was observed following pectolinarigenin treatment (*p* < 0.001; Figure 3B), indicating that both TT-DCM and pectolinarigenin impair long-term growth potential of MDA-MB-231 cancer cells.

### 2.4. TT-DCM and Pectolinarigenin Induce G0/G1 Accumulation in MDA-MB-231 Cells

**Figure 4:**
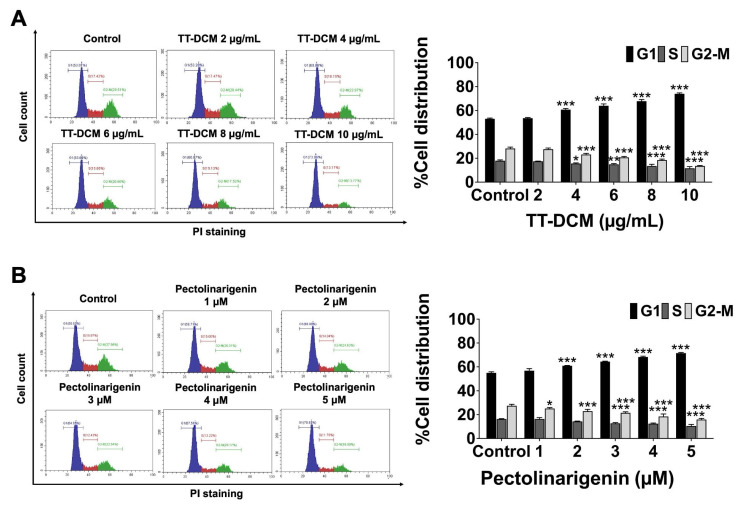
Effect of TT-DCM and pectolinarigenin on cell cycle distribution in MDA-MB-231 cells. The cells were incubated with TT-DCM (0–10 µg/mL; (**A**)) or pectolinarigenin (0–5 µM; (**B**)) for 24 h, and stained with propidium iodide (PI) for flow cytometry analysis. Bar graphs represent the percentage of cells in G0/G1, S and G2/M phases. Results are presented as mean ± S.D. from three independent experiments. Statistical significance is indicated as * *p* < 0.05, ** *p* < 0.01, and *** *p* < 0.001.

The distribution of cell cycle was examined by PI staining followed by flow cytometry after 24 h treatment. TT-DCM increased the proportion of cells in G0/G1 in a concentration-dependent manner (*p* < 0.001), accompanied by reductions in S phase and G2/M populations (*p* < 0.001; Figure 4A). Pectolinarigenin produced a similar pattern, with significant G0/G1 accumulation and concomitant decreases in S and G2/M phases (*p* < 0.001; Figure 4B). The results indicate that both TT-DCM and pectolinarigenin inhibit cell cycle progression via arresting of G0/G1 phase.

### 2.5. TT-DCM and Pectolinarigenin Promote Apoptosis in MDA-MB-231 Cells

**Figure 5:**
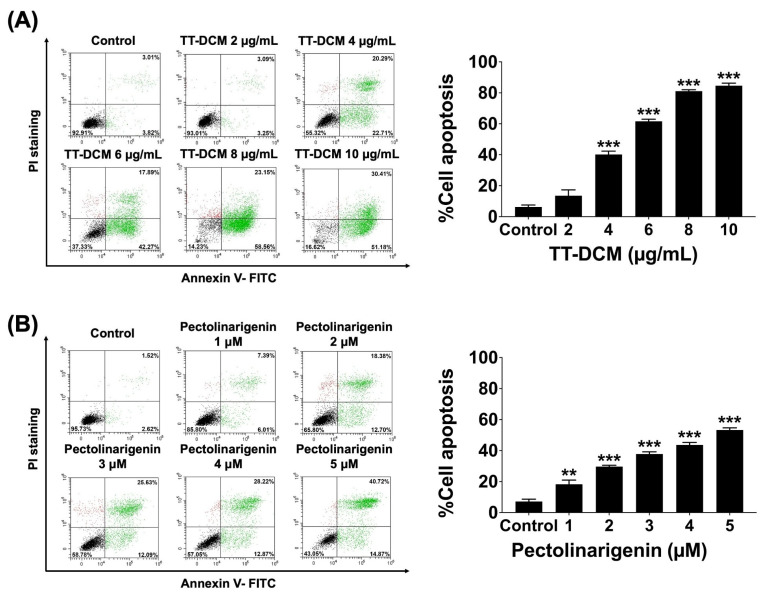
Effect of TT-DCM and pectolinarigenin on apoptosis in MDA-MB-231 cells. The cells were incubated with TT-DCM (0–10 µg/mL; (**A**)) or pectolinarigenin (0–5 µM; (**B**)) for 48 h, followed by Annexin V-FITC/propidium iodide (PI) co-staining for flow cytometry. Bar graphs show the percentage of apoptotic cells. Results are presented as mean ± S.D. from three independent experiments. Statistical significance is indicated as ** *p* < 0.01, and *** *p* < 0.001.

Apoptosis was quantified using Annexin V-FITC/PI staining followed by flow cytometry after 48 h treatment. TT-DCM treatment significantly augmented the apoptotic cells proportion in a dose-dependent manner (*p* < 0.001; Figure 5A). Pectolinarigenin likewise elevated apoptotic populations in MDA-MB-231 cells (*p* < 0.001; Figure 5B). It can be inferred that growth inhibition by TT-DCM and pectolinarigenin is associated with induction of apoptotic cell death.

### 2.6. TT-DCM and Pectolinarigenin Downregulate G1/S Regulatory Proteins

**Figure 6:**
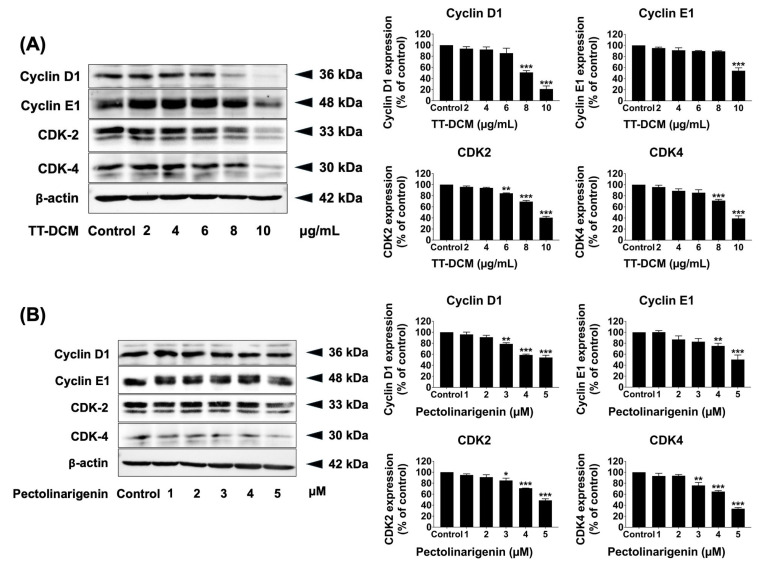
Effects of TT-DCM and pectolinarigenin on G1/S cell cycle regulatory proteins in MDA-MB-231 cells. The cells were incubated with TT-DCM (0–10 µg/mL; (**A**)) or pectolinarigenin (0–5 µM; (**B**)) for 24 h prior to protein extraction. The cyclin D1, cyclin E1, CDK2, and CDK4 protein expression levels of were determined using Western blotting, and band intensities were quantified using ImageJ software v.1.410. Results are presented as mean ± S.D. from three independent experiments. Statistical significance is indicated as * *p* < 0.05, ** *p* < 0.01, and *** *p* < 0.001.

To investigate molecular correlates of the observed G0/G1 accumulation, key regulators of G1/S transition were examined by Western blot following 24 h treatment. TT-DCM decreased cyclin D1, cyclin E1, CDK4, and CDK2 protein levels in a concentration-dependent manner (*p* < 0.001; Figure 6A). Pectolinarigenin similarly reduced the expression of these cell cycle regulatory proteins across the tested concentrations (*p* < 0.001; Figure 6B). Collectively, these data support that TT-DCM and pectolinarigenin disrupt G1/S checkpoint control in MDA-MB-231 cells, consistent with the flow cytometry results.

### 2.7. TT-DCM and Pectolinarigenin Impair Mitochondrial Membrane Potential and Modulate Apoptosis-Related Proteins

**Figure 7:**
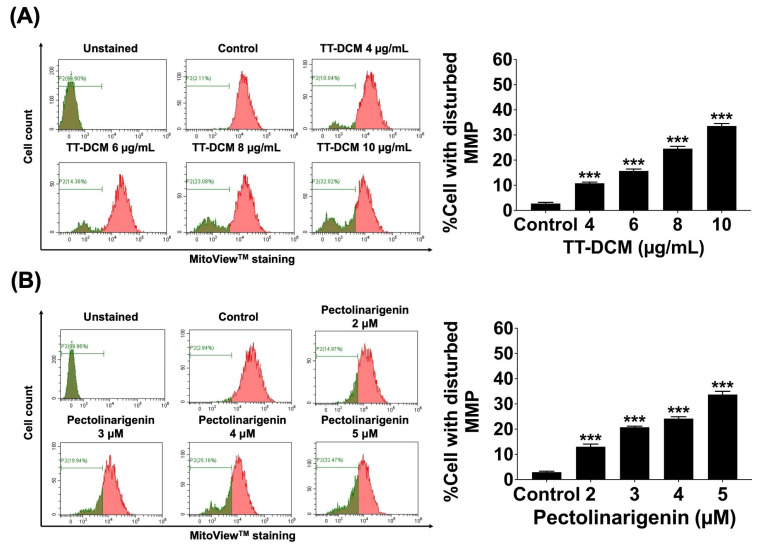
Effects of TT-DCM and pectolinarigenin on mitochondrial membrane potential (ΔΨm) in MDA-MB-231 cells. MDA-MB-231 cells were incubated with TT-DCM (0–10 µg/mL; (**A**)) or pectolinarigenin (0–5 µM; (**B**)) for 24 h, followed by assessment of mitochondrial membrane potential using MitoView^TM^ staining and flow cytometric analysis. Results are presented as mean ± S.D. from three independent experiments. Statistical significance is indicated as *** *p* < 0.001.

Mitochondrial membrane potential (ΔΨm) was assessed after 24 h exposure using MitoView^TM^ staining. TT-DCM significantly increased the proportion of cells with disrupted ΔΨm compared with the control group (*p* < 0.001; Figure 7A). Pectolinarigenin produced a comparable increase in ΔΨm disruption (*p* < 0.001; Figure 7B), indicating mitochondrial involvement in the observed cell death response.

**Figure 8:**
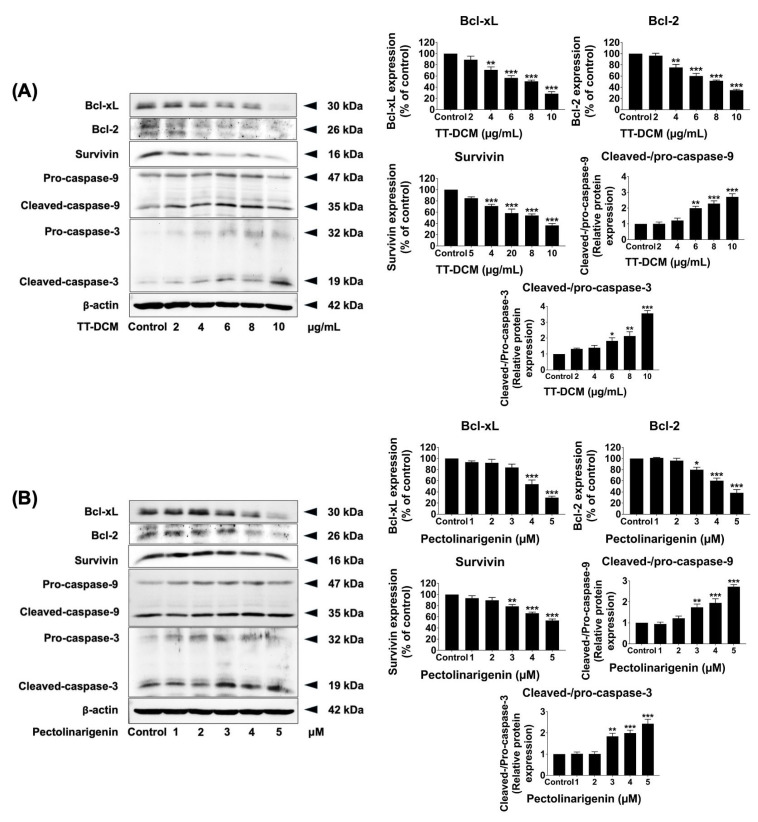
Effects of TT-DCM and pectolinarigenin on apoptosis-related protein expression in MDA-MB-231 cells. The cells were incubated with TT-DCM (0–10 µg/mL; (**A**)) or pectolinarigenin (0–5 µM; (**B**)) for 48 h prior to protein expression. Expression levels of anti-apoptotic proteins (survivin, Bcl-xl, and Bcl-2) and cleaved forms of caspase-9 and caspase-3 were evaluated by Western blotting. Band intensities were quantified using ImageJ software v.1.410. Results are presented as mean ± S.D. from three independent experiments. Statistical significance is indicated as * *p* < 0.05, ** *p* < 0.01, and *** *p* < 0.001.

To further characterize apoptosis-related signaling protein levels of Bcl-xl, Bcl-2, survivin, and cleaved-caspases-9/caspases-3 were assessed by Western blot after 48 h treatment. TT-DCM reduced Bcl-xl, Bcl-2, and surviving expressions and increased cleavage of caspase-9 and caspase-3 in a concentration-dependent manner (*p* < 0.001; Figure 8A). Pectolinarigenin elicited comparable changes (*p* < 0.001; Figure 8B). Together, these results indicate that TT-DCM and pectolinarigenin trigger apoptosis associated with mitochondrial dysfunction and caspase activation in MDA-MB-231 cells.

### 2.8. TT-DCM and Pectolinarigenin Attenuate MAPK Pathway Activation in MDA-MB-231 Cells

**Figure 9:**
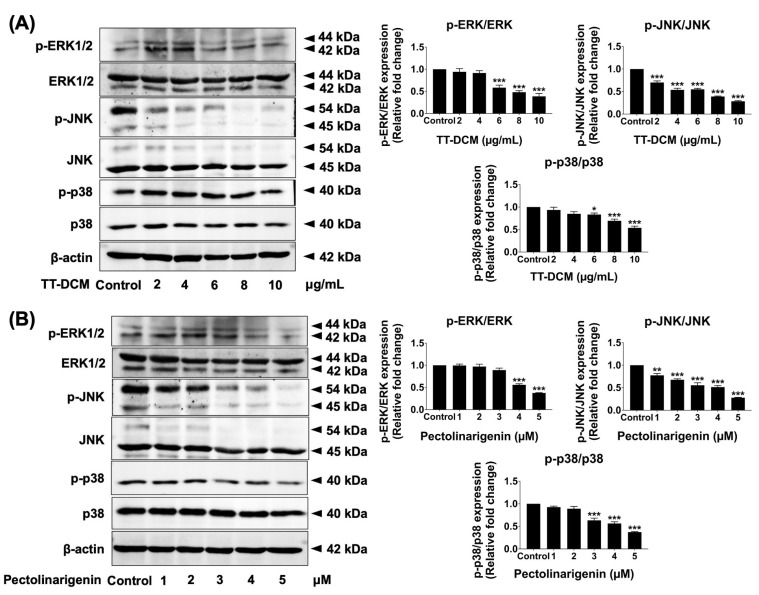
Effects of TT-DCM and pectolinarigenin on MAPK pathway activation in MDA-MB-231 cells. MDA-MB-231 cells were incubated with TT-DCM (0–10 µg/mL; (**A**)) or pectolinarigenin (0–5 µM; (**B**)) for 24 h prior to protein extraction. Phosphorylation levels of ERK, JNK, and p38 were assessed by Western blotting, with band intensities quantified using ImageJ software v.1.410. Results are presented as mean ± S.D. from three independent experiments. Statistical significance is indicated as * *p* < 0.05, ** *p* < 0.01, and *** *p* < 0.001.

To determine the contribution of MAPK signaling, phosphorylation levels of ERK, JNK, and p38 were analyzed by Western blot after 24 h treatment. TT-DCM decreased the phosphorylated form of ERK, JNK, and p38 in a concentration-dependent manner (*p* < 0.001; Figure 9A). Pectolinarigenin similarly reduced ERK, JNK, and p38 phosphorylation in MDA-MB-231 cells (Figure 9B). These findings indicate that TT-DCM and pectolinarigenin suppress MAPK pathway activation in MDA-MB-231 cells.

## 3. Discussion

Triple-negative breast cancer remains a clinically aggressive and therapeutically challenging disease entity for which effective targeted treatment options are limited. In this context, identification of bioactive natural compounds capable of modulating key oncogenic pathways represents an important area of investigation [8,9,22]. Our findings indicate that the dichloromethane fraction of *Tiliacora triandra* root (TT-DCM) and its major constituent, pectolinarigenin, suppress proliferation and promote apoptosis in MDA-MB-231 cells. Among the tested fractions, TT-DCM contained the highest level of pectolinarigenin and exhibited the strongest cytotoxic activity, supporting its selection for further mechanistic evaluation alongside the purified compound. Quantitative HPLC analysis indicated that TT-DCM contains 14.24 mg of pectolinarigenin per gram of extract (approximately 1.42% *w*/*w*). Based on this proportion, the highest tested concentration of TT-DCM (10 µg/mL) would correspond to an estimated maximal pectolinarigenin concentration of approximately 0.5 µM, which falls within the low micromolar range. Considering that purified pectolinarigenin demonstrated biological activity at similar concentrations, it is reasonable to infer that this flavonoid substantially contributes to the observed anti-proliferative effects of TT-DCM. Nevertheless, TT-DCM represents a complex phytochemical mixture, and its biological activity may result from additive or synergistic interactions between pectolinarigenin and other constituents. Therefore, the effects of TT-DCM cannot be attributed exclusively to pectolinarigenin alone.

*T. triandra* is a traditional Thai medicinal plant commonly incorporated into polyherbal formulations, including the “five roots” remedy, for the management of inflammatory and metabolic disorders [23]. Previous studies have primarily focused on ethanolic extracts derived from the leaves, reporting antioxidant activity and cytotoxic effects in cholangiocarcinoma models [24]. In contrast, the biological properties of the root—which represents a key component of traditional preparations—remain largely unexplored. Our findings therefore provide new pharmacological evidence supporting the anticancer potential of *T. triandra* root, expanding current knowledge beyond antioxidant activity and leaf-derived extracts.

Pectolinarigenin is a flavonoid distributed among various plant species and has been detected in both *T. triandra* root extracts and traditional “five roots” formulations [14]. In our MDA-MB-231 TNBC model, pectolinarigenin significantly reduced cell viability and clonogenic growth at low concentrations. In comparison with previous study, the IC_50_ value of pectolinarigenin at 48 h in MDA-MB-231 cells was 5.36 ± 1.02 μM as determined by SRB assay, which was lower than that of previous study carried out in the same cell line as determined by MTT assay (23 μM) [25]. The difference results on IC_50_ values might possibly be due to the different assay for detecting cell viability. The SRB assay provides a sensitive and reproducible measurement of cellular protein content and long-term cell viability, particularly suitable for adherent cancer cell lines and colony-forming assays. This assay is efficient and sensitive for chemotherapeutic or small molecules drug testing in various adherent cells [26,27]. Additionally, variations in cell passage number, culture conditions, compound purity, and analytical methods may further contribute to inter-study differences.

Mechanistically, TT-DCM and pectolinarigenin disrupted cell cycle progression through G0/G1 accumulation, accompanied by downregulation of cyclin D1, cyclin E1, CDK2, and CDK4. This cell cycle blockade was paralleled by induction of apoptotic cell death, characterized by mitochondrial membrane potential disruption, decreased expression of Bcl-xL, Bcl-2, and survivin, and activation of caspase-9 and caspase-3.

Previous studies have reported anticancer activities of pectolinarigenin in several tumor models, including bladder urothelial carcinoma [16], gastric cancer [28], melanoma [29], and osteosarcoma [17], with proposed mechanisms involving DNA damage/autophagy, JAK2/STAT3, and PI3K/AKT/mTOR signaling pathways. In gastric cancer and bladder carcinoma models, growth inhibition was primarily associated with G2/M arrest and autophagic responses, often observed at relatively high micromolar concentrations [16,28,30]. In contrast, our findings demonstrate that pectolinarigenin exerts potent anti-proliferative effects in MDA-MB-231 cells at substantially lower concentrations, predominantly through G0/G1 cell cycle arrest, mitochondrial dysfunction, and coordinated suppression of ERK, JNK, and p38 MAPK signaling. To our knowledge, comprehensive evaluation of MAPK pathway modulation by pectolinarigenin in triple-negative breast cancer has not been previously reported. These differences highlight tumor-specific responses to pectolinarigenin and suggest that MAPK signaling represents an important therapeutic axis in MDA-MB-231 TNBC.

Consistent with this interpretation, we observed that TT-DCM and pectolinarigenin markedly attenuated phosphorylation of ERK, JNK, and p38 MAPKs. Given the established role of MAPK signaling in regulating TNBC proliferation, survival, and therapeutic resistance, broad suppression of this pathway provides a plausible molecular basis for the observed cell cycle arrest and apoptotic responses [31,32]. Together with prior reports implicating JAK/STAT and PI3K/AKT/mTOR pathways [33,34], our findings suggest that pectolinarigenin may serve as a multitarget modulator of oncogenic signaling networks. Although our data demonstrate a clear association between MAPK pathway suppression and the observed G0/G1 arrest and apoptosis in MDA-MB-231 cells, the present study does not establish a direct causal relationship between these events. The inhibition of ERK, JNK, and p38 phosphorylation may contribute to the anti-proliferative and pro-apoptotic effects [35,36,37]; however, it remains possible that MAPK attenuation occurs as a downstream consequence of broader cellular stress responses. Additional studies employing pharmacological pathway inhibitors or rescue experiments would be required to definitively confirm MAPK signaling as the primary driver of the observed biological outcomes.

It should also be noted that the present study was conducted exclusively in the MDA-MB-231 cell model. Given the molecular heterogeneity of triple-negative breast cancer, the responsiveness of other TNBC subtypes to TT-DCM and pectolinarigenin may vary. Therefore, while our findings provide compelling evidence of anti-cancer activity in this model, further validation in additional TNBC cell lines and in vivo systems will be necessary to confirm the broader translational relevance of these results.

## 4. Materials and Methods

### 4.1. Chemicals and Reagents

Dulbecco’s Modified Eagle Medium (DMEM), penicillin–streptomycin, and fetal bovine serum (FBS) were obtained from Gibco (Grand Island, NY, USA). Modified Radioimmunoprecipitation assay (RIPA) lysis buffer, protease inhibitor cocktail, Coomassie Plus^TM^ reagent, and enhanced chemiluminescence reagents were purchased from Thermo-Fisher Scientific (Rockford, IL, USA). Sulforhodamine B (SRB), propidium iodide (PI), and anti-β-actin antibodies were obtained from Sigma-Aldrich (St. Louis, MO, USA). Annexin V-FITC apoptosis detection kits were purchased from BioLegend (San Diego, CA, USA). Primary antibodies against cyclin D1, CDK-4, CDK-2, cleaved-caspase3, cleaved caspase-9, Bcl-2, Bcl-xl, survivin, and HRP-conjugated secondary antibodies were obtained from Cell Signaling Technology (Beverly, MA, USA). Immobilon chemiluminescent substrate was purchased from Millipore (EMD Millipore, Billerica, MA, USA).

### 4.2. Preparation of T. triandra Extracts

*Tiliacora triandra* (Colebr.) Diels roots were harvested in June 2022 from Phitsanulok Province, Thailand. Plant authentication was performed at the Flora of Thailand Herbarium, Faculty of Pharmacy, Chiang Mai University (voucher No. RT045). Dried roots (500 g) were extracted with 80% ethanol (6 L) under continuous stirring for 48 h. The extract was filtered, concentrated under reduced pressure at 56 °C, resuspended in distilled water, and lyophilized to yield crude ethanolic extract (TT-EtOH) (Yield = 7.86%). For solvent fractionation, TT-EtOH (10 g) was dissolved in distilled water (500 mL), sonicated for 20 min. The extract was fully solubilized under continuous stirring. No visible precipitate remained prior to the partitioning procedure. The extract was partitioned with hexane (500 mL), then separated and evaporated. Afterward, the water fraction was partitioned with a 2:1 of dichloromethane and water fraction. This fraction was collected, evaporated, and lyophilized, and called the *T. triandra* dichloromethane fraction (TT-DCM) (Yield = 3.68%). Then, the water fraction was partitioned again with 2:1 of ethyl acetate: water fraction. The fraction was then collected, evaporated, and lyophilized and called the *T. triandra* ethyl acetate fraction (TT-EA) (Yield = 1.89%). Lastly, the residual fraction was collected, evaporated, and lyophilized, and called the *T. triandra* water fraction (TT-H_2_O) (Yield = 6.11%). TT-EtOH, TT-DCM, TT-EA and TT-H_2_O were kept at −20 °C for further experimentation.

### 4.3. Total Phenolic Content by Folin–Ciocalteu Method

Total phenolic content was quantified using a modified Folin–Ciocalteu method [38]. Extract solutions were mixed with Folin–Ciocalteu reagent followed by sodium carbonate incubation. Absorbance was recorded at 765 nm using a UV–visible spectrophotometer (Shimadzu (Kyoto, Japan) UV-1800). Gallic acid served as the calibration standard, and results were expressed as mg gallic acid equivalents per gram of extract.

### 4.4. Total Flavonoid Content by Aluminum Chloride Colorimetric Assay

Flavonoid content was assessed using an aluminum chloride colorimetric assay [39]. Extract samples were sequentially reacted with NaNO_2_, AlCl_3_, and NaOH. Absorbance was measured at 510 nm using a microplate reader. Catechin was used as the reference compound, and results were reported as mg catechin equivalents per gram of extract.

### 4.5. HPLC Analysis and Quantification of Pectolinarigenin

Pectolinarigenin in the TT-DCM fraction was identified and quantified using HPLC that carried out on a UV-Vis detector coupled with Agilent 1260 Infinity system (Agilent Technologies, Santa Clara, CA, USA). The reversed-phase Zorbax Eclipse Plus C18 column (250 mm × 4.6 mm, 5 μm particle size) with a corresponding guard column was used. The mobile phase consisted of solvent A (0.1% orthophosphoric acid in distilled water) and solvent B (acetonitrile), applied under gradient elution conditions as follows: 0–30 min, 95% A to 5% A; 30–35 min, 5% A; and 35–40 min, re-equilibration to 95% A. The flow rate was maintained at 1.0 mL/min, and detection was performed at 331 nm. Column temperature was maintained at ambient conditions [40]. An authentic pectolinarigenin standard (MedChemExpress LLC, Monmouth Junction, NJ, USA) was dissolved in methanol to prepare calibration solutions at various concentrations. A calibration curve plotting peak area versus concentration was established with good linearity within the tested range (R^2^ > 0.99). For sample preparation, TT-DCM was accurately weighed (10 mg), dissolved in 1 mL methanol, sonicated for 15 min, and filtered through a 0.45 μm membrane filter prior to injection. A 10 μL aliquot was injected for analysis.

### 4.6. Cell Culture

Human triple-negative breast cancer MDA-MB-231 cells (HTB-26™, ATCC, Manassas, VA, USA) were cultured in DMEM supplemented with 10% fetal bovine serum, penicillin (50 IU/mL), and streptomycin (50 μg/mL). Cells were maintained at 37 °C in a humidified incubator with 5% CO_2_. Cultures were passaged upon reaching approximately 70–80% confluence and used for experiments within a limited passage range. Under these conditions, MDA-MB-231 cells exhibited an average doubling time of approximately 22 h.

### 4.7. Cell Viability Test by SRB Assay

Cell viability was evaluated by the SRB assay. MDA-MB-231 cells were seeded into 96-well plates at a density of 5 × 10^3^ cells/well and allowed to attach overnight. Cells were then exposed to *T. triandra* extracts (0–100 μg/mL) or pectolinarigenin (0–50 μM) for 24 or 48 h. The cells were fixed with 10% (*w*/*v*) trichloroacetic acid at 4 °C for 1 h. After washing, cellular proteins were stained with SRB solution at 0.054% (*w*/*v*) for 30 min. Bound dye was solubilized in 10 mM Tris-buffer (pH 10.5), and absorbance was measured at 510 nm using a microplate reader (Tecan Sunrise, Männedorf, Switzerland).

### 4.8. Colony Formation Assay

The proliferative capability of MDA-MB-231 cells upon treatment was measured using a clonogenic assay. Cells were seeded in 6-well plates at a density of 500 cells/well and allowed to attach for 24 h. Subsequently, cultures were exposed to TT-DCM (0–10 μg/mL) or pectolinarigenin (0–5 μM) and maintained under standard culture conditions for 7 days, with medium refreshed every 3 days. At the end of the incubation period, colonies were fixed with 6% glutaraldehyde for 30 min and stained with toluidine blue for 15 min. Excess dye was removed by gentle washing with distilled water. Colony images were acquired using an iBright™ CL-1500 imaging system (Thermo Fisher Scientific, Waltham, MA, USA), and colony numbers were quantified. All experiments were performed in triplicate.

### 4.9. Cell Cycle Assay

MDA-MB-231 cells (1 × 10^6^ cells/well) were seeded in 6-well plates and allowed to attach overnight. To synchronize the cell cycle, cultures were serum-starved in DMEM containing 0.5% FBS for 18 h prior to treatment. Cells were subsequently exposed to TT-DCM (0–10 μg/mL) or pectolinarigenin (0–5 μM) for 24 h. Following treatment, cells were collected, washed with phosphate-buffered saline (PBS), and fixed in cold 70% methanol overnight at −20 °C. Fixed cells were then stained with propidium iodide (20 μg/mL) for 45 min in the dark. DNA content was analyzed by flow cytometry (CytoFLEX, Beckman Coulter, Brea, CA, USA), and cell cycle distribution was quantified using CytExpert DxFLEX 2.0 software.

### 4.10. Apoptosis Assay

Apoptotic cell populations were quantified using an Annexin V-FITC/propidium iodide (PI) apoptosis detection kit (BioLegend, San Diego, CA, USA). MDA-MB-231 cells (1 × 10^6^ cells per well) were seeded in 6-well plates and allowed to adhere overnight, followed by treatment with TT-DCM (0–10 μg/mL) or pectolinarigenin (0–5 μM) for 48 h. Cells were then detached using EDTA–trypsin and resuspended in binding buffer. Subsequently, samples were incubated with Annexin V-FITC (5 μL) and PI (10 μL) for 15 min at room temperature in the dark. After addition of 400 μL reaction buffer, apoptotic cells were analyzed by flow cytometry (CytoFLEX, Beckman Coulter, Brea, CA, USA) within 30 min. Data analysis was performed using CytExpert DxFLEX 2.0 software.

### 4.11. Mitochondrial Membrane Potential Assay

Changes in mitochondrial membrane potential (ΔΨm) were assessed using MitoView™ 633 dye (Biotium, Fremont, CA, USA). MDA-MB-231 cells (1 × 10^6^ cells per well) were seeded in 6-well plates and allowed to adhere overnight, followed by exposure to TT-DCM (0–10 μg/mL) or pectolinarigenin (0–5 μM) for 24 h. Cells were subsequently collected by trypsinization and incubated with 100 nM of MitoView™ 633 in serum-free DMEM for 20 min in the dark. After washing with PBS, fluorescence signals were acquired by flow cytometry at 638 nm of excitation and 660 nm of emission wavelengths. Data analysis was performed using CytExpert DxFLEX 2.0 software.

### 4.12. Western Blot Analysis

Total cellular proteins were extracted from MDA-MB-231 cells (1 × 10^6^ cells per well) using RIPA lysis buffer (100 μL per well) supplemented with protease inhibitors, followed by centrifugation at 10,000 rpm for 10 min at 4 °C. Protein concentrations were determined using the Bradford assay. Equal amounts of protein (20 μg) were separated on 12% SDS–polyacrylamide gels and electrophoretically transferred onto PVDF membranes (0.45 μm), which were pre-activated in methanol prior to transfer.

Membranes were blocked with 5% bovine serum albumin in Tris-buffered saline containing 0.05% Tween-20 (TBS-T) for 1 h at room temperature and subsequently incubated overnight at 4 °C with primary antibodies against cyclin D1, CDK2, CDK4, cleaved caspase-3, cleaved caspase-9, Bcl-xL, Bcl-2, and survivin. After washing with TBS-T, membranes were incubated with HRP-conjugated secondary antibodies for 2 h at room temperature. Protein bands were visualized using enhanced chemiluminescence reagents and captured with a chemiluminescence imaging system. β-actin served as the internal loading control. Densitometric analysis was performed using ImageJ software v.1.410.

### 4.13. Statistical Analysis

The results are expressed as mean ± standard deviation (S.D.). Statistical analyses were conducted using GraphPad Prism version 8.0. Differences between groups were evaluated using one-way analysis of variance (ANOVA) followed by Dunnett’s post hoc test, as appropriate. A *p*-value < 0.05 was considered statistically significant, with significance levels defined as * *p* < 0.05, ** *p* < 0.01, and *** *p* < 0.001.

## 5. Conclusions

This study provides the comprehensive evaluation of *Tiliacora triandra* root-derived pectolinarigenin in triple-negative breast cancer cells. TT-DCM and pectolinarigenin significantly suppressed MDA-MB-231 cell growth through coordinated regulation of cell cycle progression, mitochondrial function, and MAPK-associated signaling. Pectolinarigenin exerted anti-proliferative effects at low micromolar concentrations, inducing G0/G1 arrest, promoting apoptotic cell death, and reducing ERK, JNK, and p38 phosphorylation. These findings suggest that MAPK signaling may represent an important pathway associated with the biological activity of pectolinarigenin in this model and highlight *T. triandra* as a promising source of bioactive compounds. Further studies in additional TNBC models and in vivo systems will be required to confirm the broader therapeutic relevance of these observations.

## Figures and Tables

**Table 1:** Phytochemical study of *T. triandra* extracts.

*T. triandra* Extracts (ext.)	Total Phenolic Content (mg GAE/g ext.)	Total Flavonoid Content (mg CE/g ext.)	Pectolinarigenin Content (mg/g ext.)
TT-EtOH	142.516 ± 3.26	53.124 ± 2.88	2.48 ± 0.64
TT-DCM	183.23 ± 26.82	101.14 ± 5.91 ^a^	14.24 ± 2.32 ***
TT-EA	231.40 ± 28.86 ***	110.95 ± 5.34 ^a^	ND
TT-H_2_O	124.45 ± 22.23	50.11 ± 6.27	ND

ND: not detected. *** *p* < 0.001 vs. other *T. triandra* ext. and ^a^ *p* < 0.001 vs. TT-EtOH and TT-H_2_O extracts using one-way analysis of variance (ANOVA) followed by Dunnett’s post hoc test. Data are presented as mean ± S.D. values of three independent experiments.

## Data Availability

The original contributions presented in this study are included in the article/Supplementary Materials. Further inquiries can be directed to the corresponding author.

## Supplementary Materials

The following supporting information can be downloaded at: https://www.mdpi.com/article/10.3390/ph19030384/s1; Figure S1: HPLC identification and quantification of pectolinarigenin in the TT-DCM fraction of *Tiliacora triandra* root.
