# Supplementary material for: Pectolinarigenin from Tiliacora triandra Exhibits Potent Anticancer Activity in Triple-Negative Breast Cancer Cells Through Cell Cycle Arrest, Apoptosis, and MAPK Signaling Inhibition
Source: Pharmaceuticals (Basel). 2026 Feb 27;19(3):384. doi: 10.3390/ph19030384 (PMC13029613; doi:10.3390/ph19030384)
Supplement: Supplementary file 1 [file pharmaceuticals-19-00384-s001.zip › pharmaceuticals-4167925-supplementary.pdf]

Pectolinarigenin from *Tiliacora triandra* Exhibits Potent Anticancer Activity in Triple-Negative Breast Cancer Cells Through Cell Cycle Arrest, Apoptosis, and MAPK Signaling Inhibition

Supplementary data

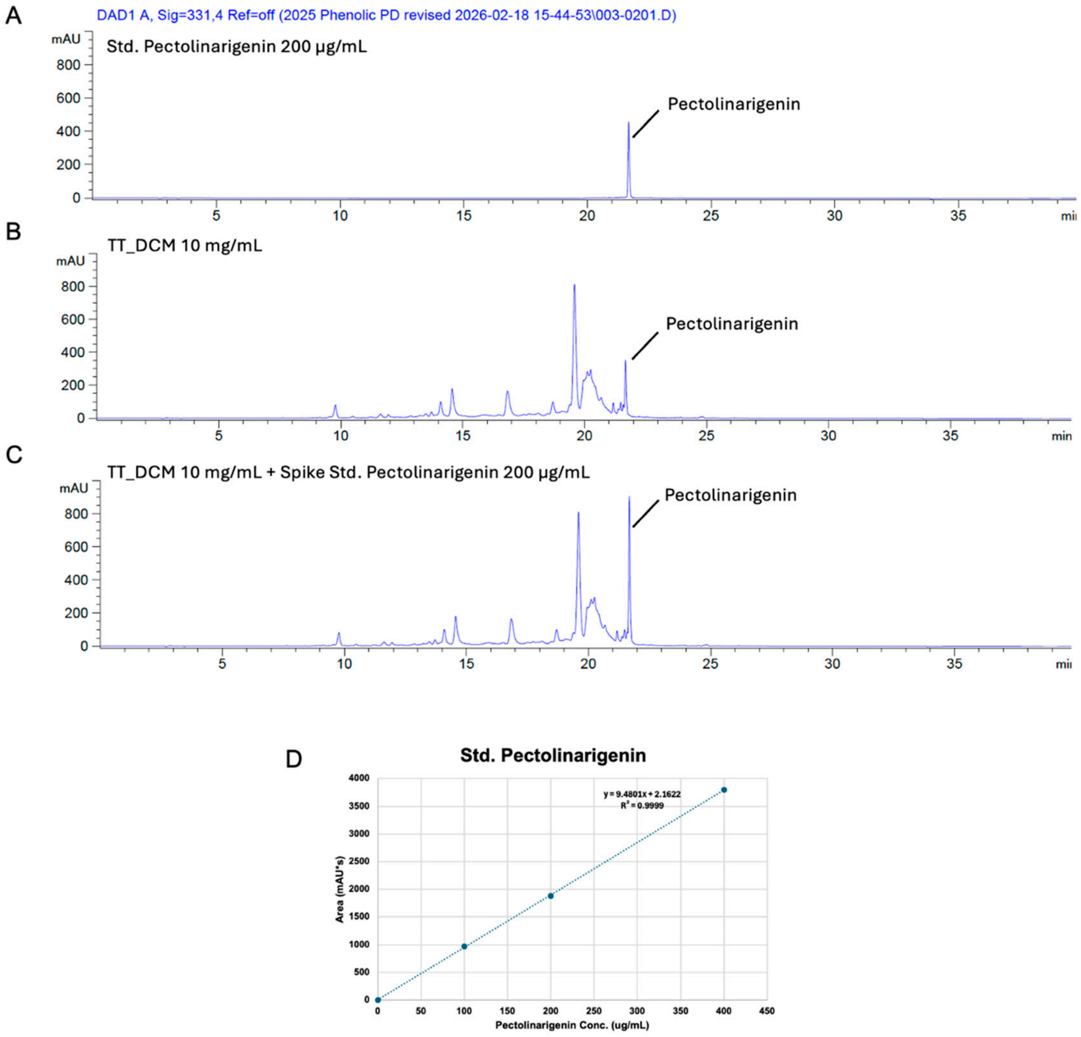

Figure S1: HPLC identification and quantification of pectolinarigenin in the TT-DCM fraction of *Tiliacora triandra* root. **(A)** Representative HPLC chromatogram of authentic pectolinarigenin standard (200 µg/mL) analyzed at 331 nm, showing a single well-resolved peak at the characteristic retention time. **(B)** HPLC chromatogram of TT-DCM (10 mg/mL), demonstrating the presence of a peak corresponding to pectolinarigenin at the same retention time as the reference standard. **(C)** Co-injection chromatogram of TT-DCM (10 mg/mL) spiked with pectolinarigenin standard (200 µg/mL), resulting in a single intensified peak without evidence of peak splitting, confirming peak identity. **(D)** Calibration curve of pectolinarigenin standard (0–400 µg/mL) constructed by plotting peak area versus concentration. Linear regression analysis yielded the equation $y = 9.4801x + 2.1622$ with excellent linearity ($R^2 = 0.9999$), supporting accurate quantification.
